# *Saussurea lappa* Exhibits Anti-Oncogenic Effect in Hepatocellular Carcinoma, HepG2 Cancer Cell Line by Bcl-2 Mediated Apoptotic Pathway and Mitochondrial Cytochrome C Release

**DOI:** 10.3390/cimb43020079

**Published:** 2021-09-08

**Authors:** Amal A. Alotaibi, Asmatanzeem Bepari, Rasha Assad Assiri, Shaik Kalimulla Niazi, Sreenivasa Nayaka, Muthuraj Rudrappa, Shashiraj Kareyellapa Nagaraja, Meghashyama Prabhakara Bhat

**Affiliations:** 1 Department of Basic Health Sciences, College of Medicine, Princess Nourah Bint Abdulrahman University, Riyadh 11671, Saudi Arabia; AMAAlotaibi@pnu.edu.sa (A.A.A.); RAAssiri@pnu.edu.sa (R.A.A.); 2 Department of Preparatory Health Sciences, Riyadh Elm University, Riyadh 12611, Saudi Arabia; 3 Department of Studies in Botany, Karnatak University, Dharwad 580003, India; sreenivasankud@gmail.com (S.N.); rmuthuraj20@gmail.com (M.R.); rajscbz@gmail.com (S.K.N.); meghubhat09@gmail.com (M.P.B.)

**Keywords:** *Saussurea lappa*, HepG2, anticancer, apoptosis, cytochrome C release, Bcl-2

## Abstract

*Background and Objectives*: *Saussurea lappa (S. lappa)* is an important species of the Asteraceae family with several purposes in traditional medicine. This study intended to explore the cytotoxic effect of *S. lappa* on HepG2 cancer cell proliferation. *Materials and Methods:* The effects of an *S. lappa* n-butanol extract on the induction of apoptosis were investigated by flow cytometry and mitochondrial cytochrome C-releasing apoptosis assay. Additionally, real-time PCR was employed to confirm apoptosis initiation. Further, qualitative estimation of the active constituent of *S. lappa* was done by gas chromatography–mass spectroscopy (GC–MS). *Results*: The cell viability study revealed that the n-butanol extract of *S. lappa* demonstrated potent cytotoxicity against HepG2 cancer cells, with an IC50 value of 56.76 μg/mL. Cell morphology with dual staining of acridine orange (AO)-ethidium bromide (EB) showed an increase in orange/red nuclei due to cell death by *S. lappa* n-butanol extract compared to control cells. Apoptosis, as the mode of cell death, was also confirmed by the higher release of cytochrome C from mitochondria, the increased expression of caspase-3 and bax, along with down regulation of Bcl-2. *Conclusion:* These findings conclude that *S. lappa* is a cause of hepatic cancer cell death through apoptosis and a potential natural source suggesting furthermore investigation of its active compounds that are responsible for these observed activities.

## 1. Introduction

Hepato-cellular cancer (HCC) is one of the most prevalent lethal malignancies, accounting for 626,000 new cases per year worldwide [1]. The five-year survival rate of HCC patients after surgery is about 20–30%. It is frequently diagnosed at an advanced stage with metastasis or with progression after locoregional therapy and has a high mortality rate, owing to the underlying liver disorder, and there is paucity of effective therapeutic options [2,3]. Up to now, sorafenib, which inhibits multiple receptor tyrosine kinases (RTKs), especially VEGF-R2/3 (vascular endothelial growth factor-receptor), platelet-derived growth factor-β (PDGFR-β), and raf kinase, is the only useful chemotherapeutic agent in managing unresected HCC [4]. It increases the average survival time by three months in patients with late-stage HCC. The common adverse effects seen with sorafenib therapy are diarrhea, fatigue, weight loss, and hand–foot syndrome. Although it lengthens the median survival time with limited side effects in these patients, the development of resistance to sorafenib is the bottleneck in extending the overall survival time for HCC patients along with its high cost, which are significant restrictions on its use [5]. Therefore, to discover novel drugs is an urgent work for the treatment of HCC.

For a long time, natural products have served as important sources of novel lead structures for the discovery of anticancer agents as their diverse ‘drug-like’ structure and ‘biologically-friendly’ molecular qualities [6,7]. Nearly 73 clinically-approved anticancer drugs are plant-derived agents, according to a recent analysis, which includes some established agents, such as vinblastine, etoposide, paclitaxel, and topotecan, which are used in lung cancers, advanced breast cancer, ovarian carcinoma, testicular tumors, and Hodgkin’s and other lymphomas [8].

*Saussurea lappa* (*S. lappa*), belonging to the family Asteraceae and is one of the best-known species within its genus; it is a perennial, aromatic, and medicinal plant [9]. It is inherent to India and Pakistan and has been farmed in Southwest China. Its dried root has been utilized since ancient times as a traditional remedy of various illnesses and diseases, such as asthma, bronchitis, ulcer, and stomach diseases in Asia, including Korea and China [10]. Sesquiterpene lactones are found in members of the Asteraceae family that belong to class of terpenoid compounds. Terpenoids are in the form of several oxygen-containing derivatives, including alcohols, aldehydes, carboxylic acids, ketones, esters, and glycosides, which consist of numerous isoprene structural units such as monoterpene (C10), sesquiterpene (C15), diterpene (C20), triterpene (C30), tetraterpene (C40), and polyterpene (C > 40). They have good cytotoxic activity and have the potential to be leading compounds to innovate efficient and safe anticancer drugs [11].

Perillyl alcohol is a monocyclic monoterpene mainly present in the essential oils of medicinal plants, and it displayed high cytotoxic activity against OVCAR-8, HCT-116, and SF-295 human tumor cell lines using the MTT assay. The cell growth inhibition percentage values were 90.92–95.82% [12]. Research has shown that ursolic acid activates cell apoptosis in prostate cancer through rho-associated protein kinase/phosphatase and tensin homolog-mediated mitochondrial translocation of cofilin-1 [13]. Cucurbitacin is a class of tetracyclic triterpenoids that also have antitumor activity [14]. Zhu et al. demonstrated that furanodiene, which is a terpenoid isolated from *Rhizoma curcumae*, produced anticancer effects through ROS production, anti-angiogenesis, apoptosis induction, and DNA strand breaks in MCF-7 human breast cancer cells and JF 305 pancreatic cell lines when transplanted into zebrafish [15].

The terpenoids can also be sub-divided into several main classes including Germacranolide, Guaianolides, Eudesmanolides, Heliangolides, etc. [16] The chief and bioactive components of *Saussurea lappa* are found to be sesquiterpenes and sesquiterpene lactones, including costunolide and dehydrocostus lactone, which belong to the germacranolides series and are described to produce cell death in some tumor cells [17,18] and therefore can be effective against HCC cell lines.

Earlier in vitro cell culture studies have shown that *S. lappa* has antiulcer, anti-inflammatory [19], and anti-hepatitis B virus activity, which is very important for the prevention of liver cancer, as well as hepatoprotective activity [20]. Additionally, *S. lappa* inhibits the growth of several types of cancer cells of neuroblastoma [21], lung cancer [22], breast cancer [23], and prostate cancer [24]. Therefore, *S. lappa* extract and its main bioactive constituents could be potential drug candidates for the treatment of HCC. In this study, we investigated the in vitro anti-cancer property of *S. lappa* extract against the hepatocellular carcinoma cell line, HepG2, along with its mechanism of cell death and qualitative estimation of its active constituent.

Objectives of the study:

The main aim of the study was to evaluate the anticancer potential of the root of *Saussurea lappa* extract against hepatocellular carcinoma using the in vitro method and characterization of possible bioactives from the root of *S. lappa.* Therefore, the following aspects of the root of *S. lappa* were planned and investigated:

(a) Determination of various pharmacognostical parameters of the root of *S. lappa* including preparation of chloroform, n-butanol, and ethyl acetate extract fractions from the root of *S. lappa* and their physicochemical testing.
(b) Gas chromatography–mass spectroscopy (GC–MS) analysis of the different extracts.
(c) Evaluation of the anticancer activity of n-butanol *S. lappa* extract by employing the in vitro cytotoxic assay method (MTT assay) against HepG2 cell lines.
(d) Exploring mechanism of action of n-butanol *S. lappa* extract against HepG2 cell lines using double staining with acridine orange (AO)-ethidium bromide (EB), the cytochrome C release apoptosis assay, and gene expression studies.

## 2. Materials and Methods

Ethics approval was granted before the start of the study by the Institutional Review Board of Princess Nourah bint Abdulrahman University with IRB log number, 19-0276.

### 2.1. Collection and Preparation of Plant Root Extract

**Figure 1:**
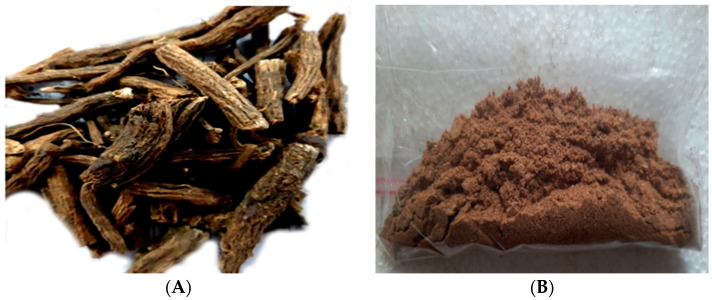
Images of (**A**) *Saussurea lappa* root and (**B**) coarse powder.

*Saussurea lappa* roots were collected from the local herbal market Dharwad, Karnataka, India and processed to remove the impurities. Plant authentication was confirmed by the Department of Botany, Karnatak University Dharwad, Karnataka, India. Roots (Figure 1A) were air dried at 40–50 °C in a hot air oven for 6–7 h and reduced to coarse powder (Figure 1B) by using a grinder and kept in a well closed air tight container for further use. The root extracts were prepared by using 50 g of powdered root in 100 mL chloroform, n-butanol, and ethyl acetate using a soxhlet apparatus, and the temperature was maintained at 60 °C for 6 h, and with water using the hot extraction method at 80 °C for 6 h. After removing the biomass residues by filtration, pooled extracts were concentrated on a rotary vacuum evaporator and further dried using an oven at 60 °C, except for the water extract, and each obtained extract was stored in a tightly closed bottle for further use [25].

### 2.2. Phyto-Chemical Analysis

The aqueous, chloroform, n-butanol, and ethyl acetate solvents were investigated for the presence of various phyto-constituents. Each stock solution was prepared by dissolving 10 mg of powder in 100 mL of the respective solvents, subjected to solvent extraction, and kept in a rotary shaker at 120 rpm for 48 h. Then extracts were air dried and stored for further usage. The crude extracts were re-suspended in their respective solvents, and qualitative tests were carried out for the alkaloids, flavonoids, carbohydrates, glycosides, tannins, proteins, resins, steroids, and triterpenoids as per standard protocols [26,27].

### 2.3. GC–MS Analysis of S. lappa Root Extracts

The qualitative analyses of *S. lappa* chloroform, n-butanol, and ethyl acetate root extracts were carried out by GC–MS analysis using a Perkin Elmer instrument (GCMS-QP2010 SE, Shimadzu Instrument, Columbia, MD, USA). The prepared root extracts of chloroform, n-butanol, and ethyl acetate were each added with 100 μL of derivatization reagent (80 μL BFSTA + 20 μL TMCS) and incubated at 65 °C for 1 h. Then each extract was injected in a Rtx5MS-30 m column with 0.25-mm ID and 0.25 μm df. The temperature maintained for injection was 300 °C, with an interface temperature at 300 °C, and an iron source maintained at 250 °C. Helium gas was used as the carrier gas at a flow rate of 1 mL min^−1^. The analysis was performed by isothermal heating for 1 min at 100 °C followed by heating at 300 °C for 20 min. The mass spectra were recorded at 2 scan sec^−1^ with a scanning range of 40 to 850 *m*/*z*. Components were quantified based on peak areas and normalization based on the internal standard using Turbo mass software [28]. The best appropriate and promising GC–MS results displaying maximum numbers of compounds were found in the n-butanol fraction. It was therefore selected for further anti-oxidant and anti-cancer studies against the hepato-cellular cell line, HepG2, considering that research of n-butanol *S. lappa* root extract on HCC and its mechanism of anticancer activity are scarce. Additionally, qualitative estimation of the active constituent has yet to be demonstrated.

### 2.4. Anti-Oxidant Assays

#### 2.4.1. DPPH (2,2-diphenyl-1-picrylhydrazyl) Free Radical Scavenging Assay

The antiradical assays of *S. lappa* n-butanol root extract were measured by DPPH free radical scavenging assay. In brief, 3 mL of DPPH solution (#RM2798-1G, DPPH, Himedia, Mumbai, India) (0.003% DPPH in methanol) was mixed with 1 mL of different concentrations (20, 40, 60, 80, and 100 µg/mL) of extract and ascorbic acid as separate standards. The tubes were kept in the dark for 30 min at room temperature and then measured for optical density at 517 nm using a UV–Vis spectrophotometer. The absorbance of standard and control was also noted, while ascorbic acid was used as standard reference. The DPPH radical scavenging assay was calculated by using the following formula:

Scavenging activity (%) = (A_0_ − A_1_)/A_0_ × 100

where A_0_ = Absorbance of DPPH and A_1_ = Absorbance of the sample.

#### 2.4.2. Ferrous Reducing Antioxidant Capacity Assay (FRAC)

The method is based on the reducing power of *S. lappa* root extract. First, 2 mL of extract was mixed with 2 mL of 2 M sodium phosphate buffer (pH 6.6) and 3 mL of 1% potassium ferricyanide. Then, the mixture was incubated at 50 °C for 20 min. Next, 3 mL of 10% trichloroacetic acid (TCA) was added to mixture and then centrifuged at 4500 rpm for 10 min. The supernatant solution (3 mL) was mixed with distilled water (3 mL) and ferric chloride (1 mL) to measure absorbance at 700 nm [29].

### 2.5. Anticancer Activity Assessment of S. lappa Root Extract

#### 2.5.1. Cell Culture

The HepG2-Human hepatocellular adenocarcinoma cancer cell lines were obtained from NCCS Pune, India. Cell lines were cultured in DMEM (Dulbecco’s modified Eagle’s medium) low glucose medium (#AL149, Himedia, Mumbai, Indai) supplemented with 10% fetal bovine serum (FBS), penicillin (100 IU/mL), streptomycin (100 μg/mL), and amphotericin-B (5 μg/mL) in a humidified incubator of 5% CO_2_ at 37 °C till confluence was produced. These cell lines in the exponential growth period were washed, dissociated with trypsin (0.2%), and resuspended in complete culture media.

#### 2.5.2. In Vitro Cytotoxic Assay (MTT Assay) Method

A 200 µL cell suspension was taken in 96 well plates at the required cell density (20,000 cells per well) and incubated at 37 °C and 5% CO_2_ for 24 h, during which a partial monolayer formed. Then, cells were treated with n-butanol root extract of different concentration (12.5 µg/mL, 25 µg/mL, 50 µg/mL, 100 µg/mL, and 200 µg/mL), while control wells, which were untreated cells, received only maintenance medium, and positive control received camptothecin (#C9911, SigmaAldrich, St. Louis, MO, USA). The plates were incubated at 37 °C in a humidified atmosphere with 5% CO_2_, 75% relative humidity for 24 h (Healforce, China). Then, 50 μL of MTT labeling compound was added, followed by incubation for 4 h. Then 100 μL of DMSO was added to each well to dissolve the formazan format on the last step. Using a microplate (ELISA) reader (ELX-800, BioTek, Vadodara, India) at wavelength 570 nm, the absorbance of the samples was measured and the IC50 value is calculated by using a linear regression equation, i.e., Y = Mx + C, where Y = 50, and M and C values were derived from the viability graph. Three independent experiments were performed.

The influence of the extract of *S. lappa* on the viability was determined using the following formula:

% viability = (A570 of treated cells − A570 of blank cells)/(A570 of controlled cells − A570 of blank cells) × 100.

The formula for calculation of the percentage of cytotoxicity [30]:

% cytotoxicity = 100 − % cell viability.

### 2.6. Apoptosis Assessment with Acridine Orange–Ethidium Bromide Staining

The n-butanol root extract of *S. lappa* was directed to check the rate of cell death in the HepG2 cancer cell line. The stains used to conduct nuclear morphological changes were acridine orange (AO) and ethidium bromide (EB). The HepG2-human hepatocellular adenocarcinoma cancer cell lines were obtained from NCCS Pune. The cultured cells were taken in a 12-well plate above the sterile coverslips coated with poly L-ornithine solution at a density of 2 × 105 cells/2 mL and incubated in a CO_2_ incubator overnight at 37 °C for 24 h. Then 56.76 µg/mL of the root extract was treated to the culture medium for 24 h and not the untreated well. After incubation, the plates were washed with PBS buffer and the PBS was removed; then the coverslip was removed and the cell plate was washed with 1 mL 1X DPBS. The cells were stained with 200 µL staining solution for 10 min and observed under fluorescence microscope with a filter cube with excitation of 560/40 nm and emission of 645/75 nm for EtBr and excitation of 470/40 and emission of 525/50 for acridine orange [31]. The images were taken from Image J Software v1.48.

### 2.7. Cytochrome C Releasing Apoptosis Assay

The cytochrome C releasing apoptosis assay provides an effective means for detecting cytochrome C translocation from mitochondria into the cytosol during apoptosis [32]. The n-butanol root extract of *S. lappa* was directed to check the cytochrome C releasing apoptosis assay in our target cell line. The cultured cells were taken in a 6-well plate at a density of 3 × 105 cells/2 mL and incubated in a CO_2_ incubator overnight at 37 °C for 24 h. Then, 56.76 µg/mL of the root extract was treated to the culture medium and not the untreated well, followed by incubation for 24 h. At the end of the treatment, the cells were harvested directly into 12 × 75 mm polystyrene tubes. The cells were centrifuged for five minutes at 300× *g* at 25 °C, and then the supernatant was removed carefully. The cells were fixed and permeabilized in 70% pre-chilled absolute ethanol and stored in −20 °C for 30–40 min. The suspension was centrifuged again at 300× *g* at 25 °C, and ethanol was removed. The cells were washed twice with PBS to remove excess ethanol, and the PBS was decanted completely. The cells were stained with 10 μL of cytochrome C-conjugated FITC antibody (Invitrogen) for 20 min in the dark at RT. The cells were re-suspended in 200 μL of PBS and analyzed by flow cytometry using the FL-1 channel at excitation of 488 nm and emission wavelength of 520 nm, respectively, to analyze cellular distributions of cytochrome C [32].

### 2.8. Gene Quantification by qRT-PCR

Total RNA was isolated using a Qiagen RNeasy kit for all samples. It was treated with DNAse and purified to avoid genomic DNA contamination. RNA was quantified by UV–Vis using QIAxpert (Qiagen, Foster City, CA, USA). RNA was also run on agarose gel. The ladder used was Lambda HindIII/EcoRI. Total extracted RNA (1 μg) was reverse transcribed using an IScript cDNA synthesis kit (Bio-Rad, Foster City, CA, USA) with random hexamer + oligo dT primers as per the reaction mentioned in Table 1. The tubes were incubated in a PCR cycler with priming for 5 min at 25 degrees, RT 20 min at 46 degrees, RT inactivation 1 min at 95 degrees. The newly synthesized first strand cDNA was stored at −20 °C. The primers designed for the respective gene expression studies were as mentioned in Table 2 below. All the primers (HPLC grade) were synthesized from Eurofins, Bangalore. The relative quantification of the gene expression was done in Qiagen Rotor Gene Q 5plex HRM using the SYBR Green Chemistry (SensiFAST SYBR Hi-ROX kit, Bioline, Taunton, MA, USA). The reaction was carried out in a 25 µL reaction volume with the components as first strand cDNA—1 µL, SYBR Green Master Mix (2X)—12.5 µL, forward primer (10 µM)—1 µL, reverse primer (10 µM)—1 µL, and nuclease-free water—9.5 µL. The thermo-cycling conditions with a total number of 40 cycles were established as 5 min at 95 °C, followed by 10 s at 95 °C, 20 s at 60 °C, and 20 s at 72 °C. Each reaction was conducted in triplicate, and the 2^−ΔΔCt^ method was applied to calculate fold-change differences between control and treated samples, and GAPDH was used as an internal control.

### 2.9. Statistical Analysis

The results of each experiment were expressed as the means ± standard deviation (SD, for each group *n* = 3). Statistical significance at *p* < 0.05 between the groups was evaluated by one-way ANOVA analysis of variance.

## 3. Results

### 3.1. Phytochemical Analysis

The results of phytochemical tests confirmed that its roots are rich source of various bioactive compounds such as flavonoids, alkaloids, glycosides, coumarines, phenols and tannins, steroids, and essential oil contents. The presence of various phytochemicals was noticed qualitatively in all the four different extracts, and the maximum number of phytochemicals was identified in aqueous extracts (Table 3).

### 3.2. Identification of S. Lappa Root Components by GC–MS Analysis

**Figure 2:**
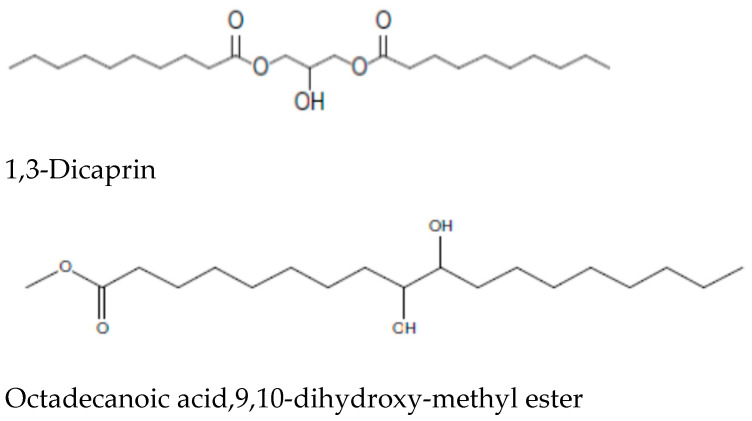

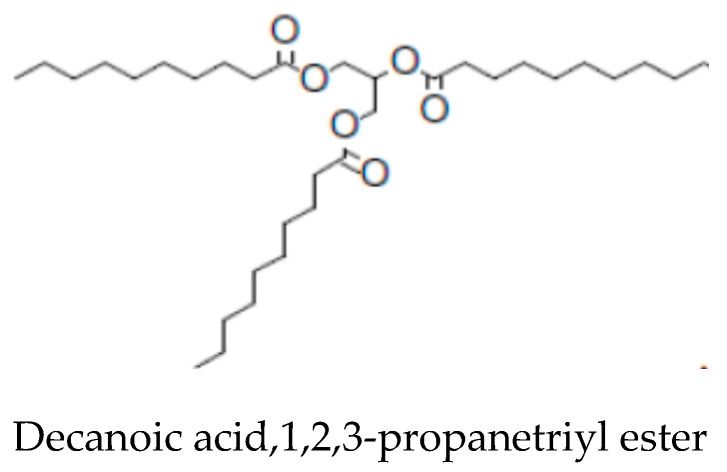
Major constituents found in n-butanol *S. lappa* root extract.

**Figure 3:**
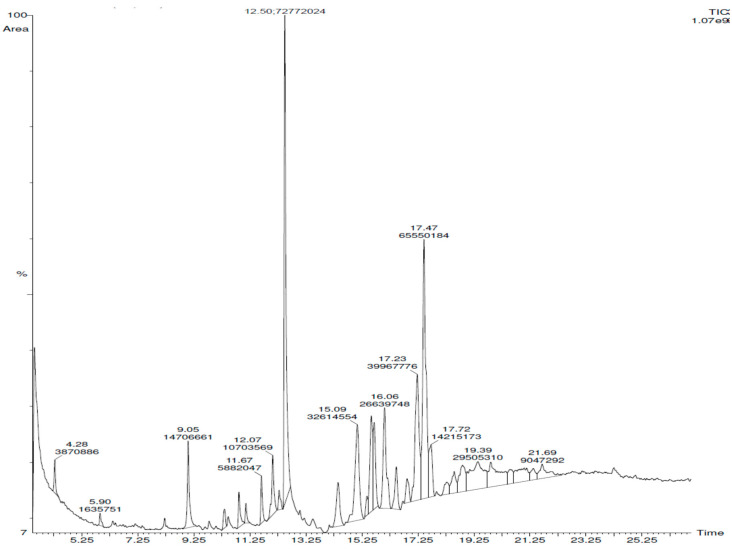
Chromatogram of n-butanol root extract of *Saussurea lappa* by GC–MS.

Figure 2 depicts the GC–MS analysis of *S. lappa* root extracts of n-butanol root extract of *Saussurea lappa*. Its chloroform, n-butanol, and ethyl acetate extracts recorded 54 compounds, among those costunolide, Bohlmann k2631, and 2(3H)-benzofuranone,6-ethenylhexahydro-6-methyl-3-methylene-7-(1-methylethenyl)-, 3S-(3α,3aα,6α,7β,7aβ)]-were present in both n-butanol and ethyl acetate extract; and 1,2-dicaprin was present in chloroform and n-butanol extract. Among the 54 compounds, the maximum (27) were seen in n-butanol extract. It was therefore selected for further anti-oxidant and anti-cancer studies. The retention time and the percentage amounts of the compositions with their molecular formula present in n-butanol root extract are displayed in (Table 4), and the chemical structures of the major constituents are displayed in Figure 3.

### 3.3. Antioxidant Assays

**Figure 4:**
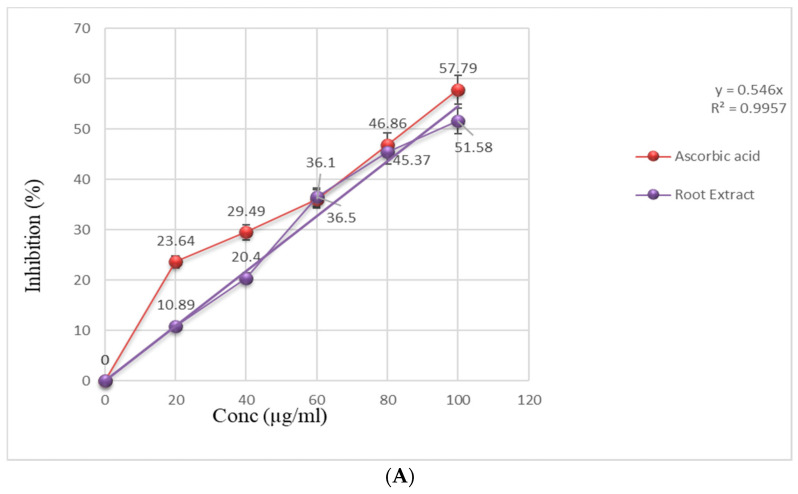

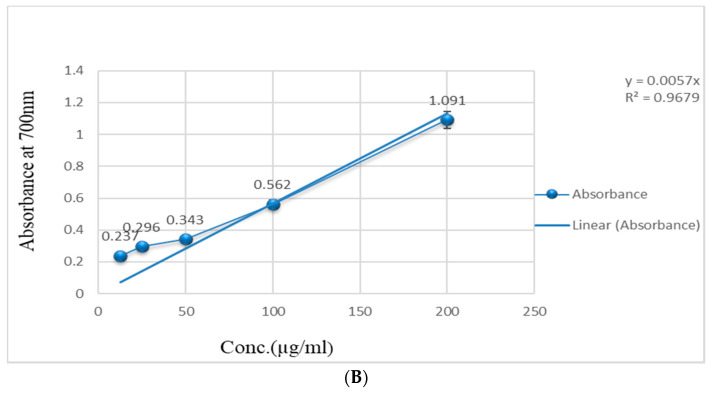
(**A**) Free radical scavenging activity of the n-butanol extracts from roots of *S. lappa* and positive controls measured in DPPH assay. (**B**) Absorbance of FRAC assay of root extract at different concentrations.

The values of the radical scavenging effect of *Saussurea lappa* n-butanol root extract and ascorbic acid are depicted in Figure 4A, where both exhibited dose dependent increased scavenging free radicals of DPPH where it converted to DPPHH, with increasing concentrations. The scavenging activity of extract was greater than 50% at concentrations of 100 µg/mL but less than that of standard ascorbic acid. In FRAC, reducing power of extract was determined by reduction of Fe^+3^ to Fe^+2^ in various concentrations of extract. The absorbance of mixture was increased with the increase in concentration of extract, indicating the reducing potential of root extract (Figure 4B).

### 3.4. Anticancer Activity of S. lappa N-Butanol Root Extract

**Figure 5:**
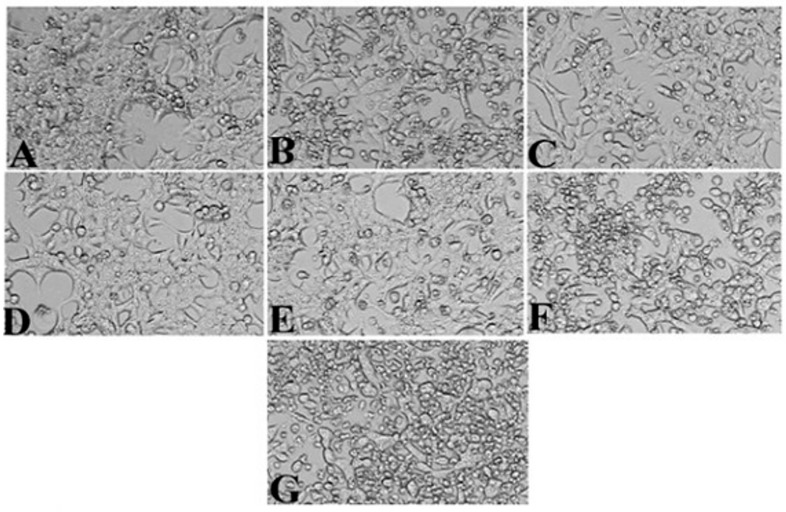
In vitro anticancer activity of root extract of *Saussurea lappa* ((**A**)—untreated, (**B**)—positive control, (**C**)—12.5 µg/mL, (**D**)—25 µg/mL, (**E**)—50 µg/mL, (**F**)—100 µg/mL, (**G**)—200 µg/mL). (the images were captured at 10× magnification by inverted biological microscope).

**Figure 6:**
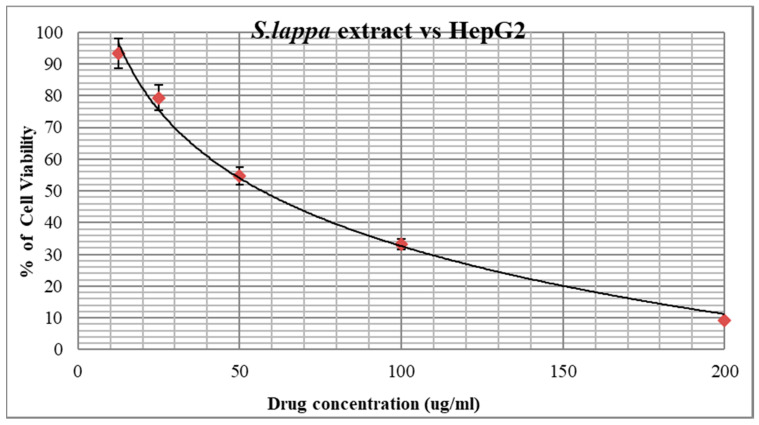
Graph showing the percentage cell viability of root extract against HepG2 cell line after 24 h.

The n-butanol root extract of *S. lappa* was treated against HepG2 cell lines for anticancer activity by MTT assay. The extract showed significant growth inhibitory potential with the IC50 value at a concentration of 56.76 µg/mL compared to the positive control, camptothecin, with an IC50 concentration at 10 µM equivalent to 3.48 µg/mL used for the study, after the treatment for 24 h of incubation at 37 °C temperature (Figure 5 and Figure 6). Thus, n-butanol root extract showed 61.36% similar potency against the HepG2 cancer cells compared to camptothecin.

### 3.5. Apoptosis Assessment with Acridine Orange–Ethidium Bromide Double Staining Assay of S. lappa N-Butanol Root Extract

**Figure 7:**
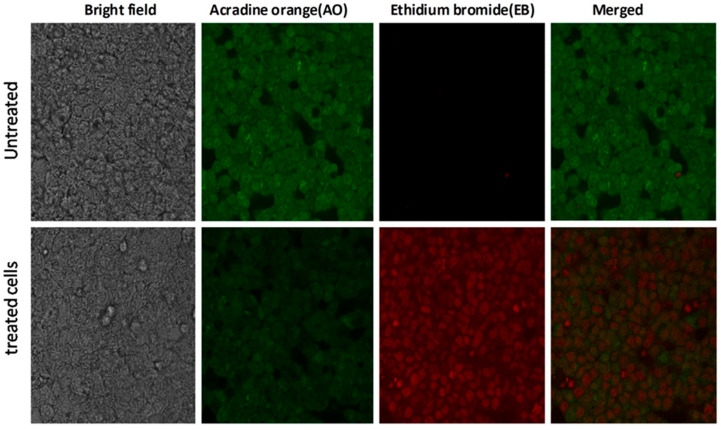
*S. lappa* n-butanol root extract-induced apoptosis. Dual staining study of HepG2 cells by acridine orange (AO) and ethidium bromide (EB) (AO represents viable cells and EB represents dead cells). Untreated and treated with n-butanol extract (56.76 µg/mL) representing the changes in nuclear morphology of cells (the images were captured at 40× magnification).

Apoptotic cell morphological analysis was carried out on untreated and *S. lappa* n-butanol root extract-treated HepG2-human hepatocellular adenocarcinoma cancer cells using the acridine orange (AO) and ethidium bromide (EB) double staining method. These are nucleic acid-binding dyes used for fluorescence of cancer cells. AO is an intercalating dye that gives a green color, which indicates the viable cells, whereas the EB produces a red color, indicating the dead cells (Figure 7). After the treatment of root extract against HepG2 cancer cells, the cells were observed under a fluorescent microscope. The *S. lappa* n-butanol extract concentration of 56.76 µg/mL produced nuclear features suggestive of apoptosis.

### 3.6. Cytochrome C Releasing Apoptosis Assay

**Figure 8:**
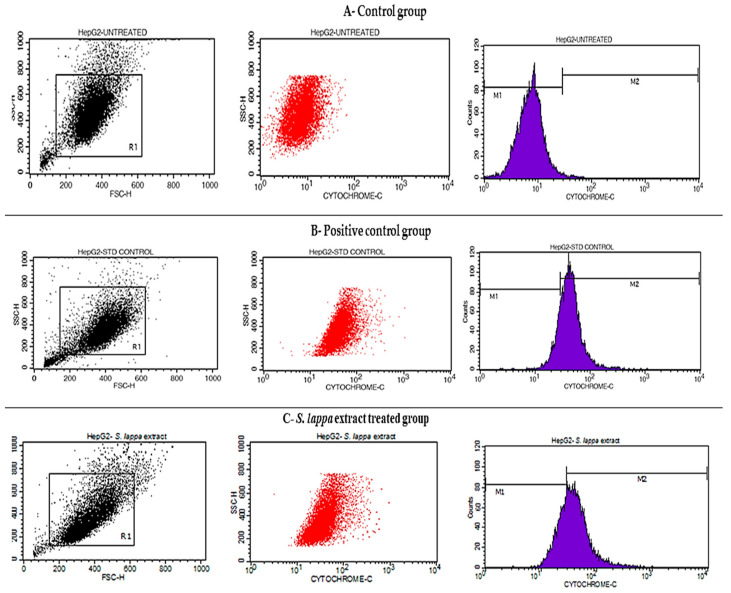
Histograms representing cytochrome C expression study with (**A**)—control, (**B**)—positive control-treated, and (**C**)—*S. lappa* n-butanol root extract treated with IC50 concentration, viz., 56.76 ug/mL against the HepG-2 cells using BD FACS Calibur, Cell Quest Pro Software (Version: 6.0).

We observed that in HepG-2 cells, the expression of cytochrome C was very low in untreated cells (0.64%) compared to camptothecin (10 μm), showing 82.89% cytochrome C expression. The *S. lappa* extract with IC50 concentration, viz., 56.76 ug/mL, showed 67.78% of cytochrome C, respectively, as depicted in Figure 8 and Table 5.

The Cyt-FITC histogram of the gated HepG2 cells distinguished cells at the M1 and M2 phases. (Here M1 refers to negative expression/region and M2 refers to the positive expression/region). Gating of M1 and M2 phases was approximate and refined using software (Cell Quest Software, Version 6.0) analysis.

### 3.7. Gene Quantification by qRT-PCR

**Figure 9:**
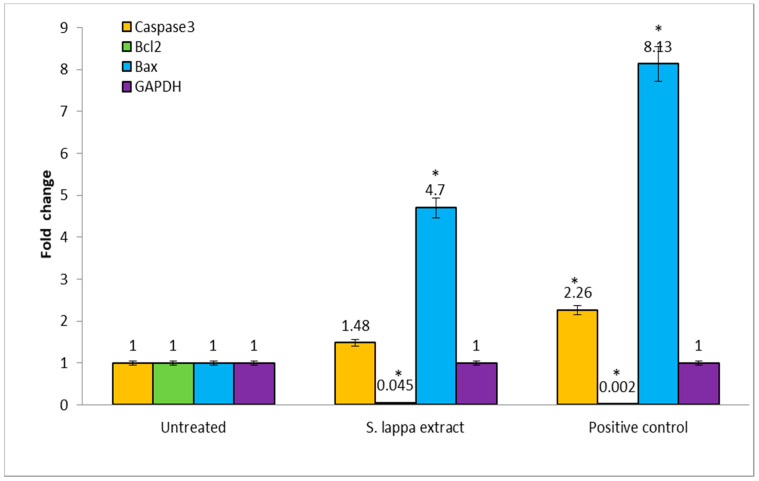
Relative mRNA expression of caspase-3, Bcl-2, Bax, and GAPDH in Untreated, *S. lappa* extract, and positive control-treated HepG2 cells by RTqPCR. The significant differences from control are indicated by * *p* < 0.05.

Our results indicated that the *S. lappa* n-butanol root extract-treated cells showed increases in the levels of apoptosis-related genes. The obtained results suggest that relative gene expression levels of caspase-3 and bax genes were up regulated and the anti-apoptotic bcl-2 gene was down regulated in treated groups compared to the untreated group as depicted in Figure 9. GAPDH was used as an internal control in the current study. These results strongly support the apoptosis induction by *S. lappa* n-butanol root extract in HepG2 cells through the up-regulation of caspase-3 and Bax genes together with a down-regulation of Bcl-2, respectively. Agarose gel electrophoresis image of isolated RNA (Figure S1), validation of primers with each cDNA (Figure S2), melting curves of genes (Figure S3) and amplification curve (Figure S4) are added in supplementary material (S1).

## 4. Discussion

Hepatocellular carcinoma (HCC) is the fourth most frequent condition of cancer fatality worldwide and the third most common cancer in men [33]. It is the second most prevalent cancer affecting Saudi males [34]. Chemotherapy is a unit of valuable treatment modalities for advanced HCC. It is employed to treat patients who are judged as being unsuitable candidates for surgical resection, local ablative therapy, or transarterial chemoembolization (TACE), which includes patients who have extrahepatic metastasis, show evidence of vascular invasion, or are refractory to TACE [35]. Regarding systemic chemotherapy, sorafenib, a small molecule that inhibits multiple receptor tyrosine kinases (RTKs), is used as the first-line setting for patients with advanced HCC because almost all HCC patients have cirrhosis, chemotherapies, or major resections are not well tolerated. Thus, new strategies for HCC therapy are crucially needed.

In recognition of nature’s potential, several plant screenings have performed as potential anticancer drug candidates, and *Saussurea lappa* is an example of this. In this study, *S. lappa* root was extracted by chloroform, n-butanol, and ethyl acetate solvent that were characterized by GC–MS in order to obtain the diversity of biologically active phyto-chemicals. A total of 54 compounds were recorded, out of which most were seen in the n-butanol extract. The results of the present study presented that the *S. lappa* root n-butanol extract exerted anti-oxidant activity along with cytotoxic effects on the HepG2-human hepatocellular adenocarcinoma cancer cell line. We also demonstrated their cell death analysis using nucleic acid-binding dyes, acridine orange and ethidium bromide, which cause fluorescence of cancer cells along with determining their effects on the mitochondrial membrane permeability by the cytochrome C-releasing apoptosis assay and regulation of activity level of genes of bax, bcl-2, and caspase-3.

The GC–MS analysis of the n-butanol *S. lappa* root extract is depicted in Figure 2. The retention time and the percentage amounts of the compositions are displayed in Table 4. 1,3-Dicaprin (17.3%), octadecanoic acid,9,10-dihydroxy-,methyl ester (11.5%), and decanoic acid,1,2,3-propanetriyl ester (9.4%) were the primary constituents (Figure 3). The remaining compounds are present in small proportions.

The anti-oxidative activities of *S. lappa* n-butanol root extract on both test systems of DPPH free radical scavenging and FRAC increased in a concentration-dependent manner (Figure 4A,B), and therefore it acts as a good source of antioxidant agent. Similar results were seen in the study conducted by Kyung-Mi Chang et al. [36] A previous study showed that costunolide, isolated from the root of *S. lappa*, is a potent inducer of apoptosis and facilitates its activation via reactive oxygen species generation in HL-60 human leukemia cells [37].

One of the main hallmarks of cancer is cell death evasion [38]. In our study, *S. lappa* n-butanol root extract displayed potent cytotoxic activity against HepG2 with an IC50 concentration of 56.76 µg/mL (Figure 5 and Figure 6). S.M. Moon et al. reported that *S. lappa* methanol extract inhibited the growth of KB human oral cancer cells in a dose- and time-dependent manner exhibiting IC50 value of 30 µg/mL, approximately [39]. This variation in IC50 values may result from usage of *S. lappa* from different geographical sources and growing conditions and having been used on various cell types. Tian et al. (2017) reported that *S. lappa* ethanol extract shows significant anticancer activity against lymph node carcinoma of the prostate (LNCaP) cancer cells [24].

In the next series of experiments, we carried out acridine orange (AO)–ethidium bromide (EB) double staining cell morphological analysis using the IC50 concentration of *S. lappa* n-butanol root extract against the HepG2 cell line of 56.76 µg/mL. Live cells stained uniformly green and can be distinguished from apoptotic cells. Early apoptotic cells will have bright green nuclei and late apoptotic cells display condensed and fragmented orange chromatin, and cells that have died from direct necrosis have structurally normal orange/red nuclei due to co-staining with AO/EB. The treatment group showed fragmented shrunken and marginated nuclei in contrast to the normal and large nucleus in the untreated cells, proving the apoptotic potential of the extract (Figure 7). Similar results were seen in a recent study [40].

In order to determine the potential mechanism by which *S. lappa* n-butanol root extract causes decreased cell viability, estimation of the cytochrome C-releasing apoptosis assay was done. Release of cytochrome C from the mitochondria to the cytosol is triggered by apoptotic stimuli. In the cytoplasm, the cytochrome C binds apoptotic protease activating factor, which activates the apoptotic initiator procaspase 9. Cytosolic cytochrome C functions in the activation of caspase 3, an ICE family molecule that is a key effector of apoptosis. Untreated HepG2 cells showed very little cytochrome C expression, since there was no apoptosis. The observed percent of cytochrome C-expressed cells in untreated, camptothecin, and 56.76 µg/mL of *S. lappa* n-butanol root extract-treated HepG2 cells were 0.64%, 82.89%, and 67.78%, respectively, as seen in Figure 8. These observations suggest to us that the *S. lappa* n-butanol root extract may have significant apoptosis potential in HepG2 cancer cells with possible therapeutic potential against liver cancer via cytochrome C-mediated apoptosis. Li. et al. demonstrated that beta-lapachone induced rapid release of cytochrome C followed by activation of caspase-3 in apoptotic cell death in numerous human carcinoma cell lines of the breast cancer cell lines MCF-7, 21 MT, 21 NT, and 21 PT; AD 2780s (human ovary carcinoma); human colon adenocarcinoma cell lines SWI116, HT-29, and DLD; human prostate tumor cells PC-3, DU145, and LNCaP; and a human lung carcinoma cell line (G480). This study concluded that beta-lapachone is a potential anti-cancer drug acting on the mitochondrial cytochrome C–caspase pathway [41].

It has been known that caspase family activation represents one of the earliest known steps in the cell death process [42]. Activation of caspase-3 is involved in the regulation of intrinsic and extrinsic apoptotic pathways [43,44]. Bcl-2 is a member of a large family of cell survival regulating proteins consisting of both pro- and anti-apoptotic regulators. The bax/bcl-2 regulation is predominant mechanism of apoptosis evasion used by cancers [45]. We therefore assessed the activity level of bax, bcl-2, and caspase-3 genes. There was relative up-regulation of pro-apoptotic caspase-3 and bax genes and down-regulation of the antiapoptotic bcl-2 gene in *S. lappa* n-butanol root extract-treated HepG2 cells compared to the untreated group (Figure 9). Ko et al. reported cytotoxic effects of *Saussurea lappa* on AGS gastric cancer cells, and they determined that there was no effect of *S. lappa* on bcl-2 expression but had strong stimulating effect on bax gene expression [40]. In line with our study, Moon et al. demonstrated that *S. lappa* extract induced the proteolytic processing of caspase-3, a significant increase of Bax, and marked reduction of Bcl-2 in KB human oral cancer cells [39].

In one of the studies, a methanolic leaf extract of *Morus alba* that contained rutin, isoquercetin, and various derivatives of kaempferol and quercetin glycosides showed inhibition (IC50 = 33.1 µg/mL) of HepG2 cells, which was achieved by activation of caspases to induce cell apoptosis and inhibition of topoisomerase II activity [46]. A study conducted by Cho et al reported the anticancer activity in HepG2 cells by isoegomaketone, an essential oil component isolated from *Perilla frutescens*, when treated for over 24 h, and cleaved caspase-3, caspase-8, and caspase-9 in a time-dependent and dose-dependent manner [47]. Additionally, Wanga et al. detailed that isoegomaketone inhibited cells and decreased Huh-7 hepatoma cell carcinoma and tumor-xenograft nude mice tumor weight and volume. Isoegomaketone in the concentration of 10 nM/L decreased pAkt without affecting Akt. Hepatoma cell carcinoma tumor growth was suppressed through PI3K/Akt signaling pathway blocking [48].

GC–MS is a useful and reliable method for the rapid identification of complex plant extracts. In our study, the GC–MS analysis of *S. lappa* n-butanol extract revealed 27 phytoconstituents including compounds such as 2(3H)-benzofuranone,6-ethenylhexahydro-6-methylene-7-(1-methylethenyl)-, **[3aS-(3aα,6α,7β,7aβ)]**, commonly known as dehydrosaussurea lactone, with another sesquiterpene lactone costunolide. These compounds have also shown an inhibitory effect against breast cancer cell growth by inducing cell cycle arrest and apoptotic action against cancerous cells, indicating their significant medicinal properties [49]. Wang, et al. showed the molecular anticancer mechanism of oridonin, a tetracyclic terpenoid that is the main active compound in *Rabdosiae rubescens* in HepG2 cancer cells by G2/cell cycle arrest and apoptosis when applied for 24 h. *R. rubescens* belongs to the family Lamiaceae and contains important chemical compounds including monoterpenes, sesquiterpene, diterpene, and terpenoids [50]. Moreover, the plant extracts’ medicinal effects could be primarily attributed to their secondary products, which act synergistically rather than as single compounds [51]. Thus, it can be concluded that the anticancer and anti-oxidant effects observed in the *S. lappa* extract are linked with the presence of these different compounds.

## 5. Conclusions

The *S. lappa* n-butanol extract demonstrated anti-proliferation capacity against the HepG2 cancer cell line successfully by inducing cell death. Our study showed the potential role of *S. lappa* in activating apoptosis causing dose-dependent increases in early and late apoptosis cell populations. Moreover, apoptosis initiation induced by *S. lappa* was confirmed by cytochrome C-mediated apoptosis through increased mitochondrial membrane permeability, causing cytochrome C release and upregulation of apoptosis gene markers in HepG2 cancer cells. This study provides preliminary data that propose *S. lappa* as a valuable source of potentially new natural anti-hepatic cancer compound(s) that act by triggering apoptotic cell death. Further research is required to find effective compounds as well as the cellular and molecular mechanisms involved.

## Figures and Tables

**Table 1:** cDNA synthesis reaction mix.

cDNA Synthesis Reaction Mix Constituents	Vol in µL
5X IScript reaction mix	10
Nuclease free water	18
RNA	10
Reverse transcriptase enzyme	2

**Table 2:** Primer sequences for quantitative real-time polymerase chain reaction (qRT-PCR).

	Gene Name	Forward Primer Sequences	Reverse Primer Sequences
**1**	Caspase 3 (210 bp)	5′-TGTTTGTGTGCTTCTGAGCC-3′	5′-CACGCCATGTCATCATCAAC-3′
**2**	Bcl-2 (141 bp)	5′-ATGTGTGTGGAGACCGTCAA-3′	5′-GCCGTACAGTTCCACAAAGG-3′
**3**	Bax (133 bp)	5′-ATGTTTTCTGACGGCAACTTC-3′	5′-AGTCCAATGTCCAGCCCAT-3′
**4**	GAPDH (113 bp)	5′-TCAAGAAGGTGGTGAAGCAG-3′	5′-AAAGGTGGAGGAGTGGGTGT-3′

**Table 3:** Phytochemical investigation of *S. lappa* root extracts.

Chemical Constituent	Aqueous Extract	Chloroform Extract	n-Butanol Extract	Ethyl Acetate Extract
Alkaloids	+	+	+	+
Steroids	+	+	+	−
Terpenoids	+	+	+	−
Flavonoids	+	+	+	+
Carbohydrates	+	+	+	+
Proteins	+	−	+	+
Phenols	+	+	+	+
Tannins	+	+	+	+
Saponins	+	+	−	−
Glycosides	+	+	+	+
Coumarins	+	−	+	+
Fixed oil	+	−	+	−

(+) Indicates the presence and (−) indicates absence.

**Table 4:** Compounds present in n-butanol root extract of *Saussurea lappa* analyzed by GC–MS.

Sl No	Retention Time	Peak Area %	Name of the Compound	Molecular Formula
1	4.289	2.8	Dimethylsulfoxonium formylmethylide	C_4_H_8_O_2_S
2	5.904	0.6	(Z)-1-Chloro-2(methylsulfonyl)ethylene	C_3_H_5_ClO_2_S
3	9.046	3.4	cis,cis,cis-7,10,13-Hexadecatrienal	C_16_H_26_O
4	10.321	0.1	l-Gala-l-ido-octose	C_8_H_16_O_8_
5	10.866	0.9	2(3H)-Benzofuranone,6-ethenylhexahydro-6-methyl-3-methylene-7-(1-methylethenyl)-, **[3aS-(3aα,6α,7β,7aβ)]-**	C_15_H_20_O_2_
6	11.111	2.6	Tricyclo [4.3.1.1(3,8)]undecane-1-carboxylic acid	C_12_H_18_O_2_
7	11.667	0.6	Bohlmann k2631	C_15_H_20_O_2_
8	12.072	0.7	Costunolide	C_15_H_20_O_2_
9	12.312	0.5	2(3H)-Benzofuranone,6-ethenylhexahydro-6-methyl-3-methylene-7-(1-methylethenyl)-, **[3aS-(3aα,6α,7β,7aβ)]-**	C_15_H_20_O_2_
10	12.502	11.5	Octadecanoic acid,9,10-dihydroxy-,methyl ester	C_19_H_38_O_4_
11	14.408	3.7	1,2-Dicaprin	C_23_H_44_O_4_
12	15.088	9.4	Decanoic acid,1,2,3-propanetriyl ester	C_33_H_62_O_6_
13	15.433	1.1	Vinyl decanoate	C_12_H_22_O_2_
14	15.588	6.8	Decanoic anhydride	C_20_H_38_O_3_
15	15.688	4.8	Decanoic acid, 2-hydroxy-1-(hydroxymethyl)ethyl ester	C_13_H_26_O_4_
16	17.234	8.8	3,5,9-Trioxa-4-phosphanonadecan-1-aminium, 4-hydroxy-N,N,N-trimethyl-10-oxo-7-[(1-oxodecyl)oxy]-, hydroxide, innersalt, 4-	C_28_H_56_NO_8_P
17	17.469	17.3	1,3-Dicaprin	C_23_H_44_O_5_
18	17.714	2.9	Estra-1,3,5(10)-trien-17′-ol	C_18_H_24_O
19	18.259	0.6	9,10-Secocholesta-5,7,10(19)-triene-3,24,25-triol, (3%,5Z,7E)-	C_27_H_44_O_3_
20	18.535	2.2	2-Bromotetradecanoic acid	C_14_H_27_BrO_2_
21	18.860	1.7	Cyclopropanetetradecanoic acid, 2-octyl-, methyl ester	C_26_H_50_O_2_
22	19.385	2.0	1-Heptatriacotanol	C_37_H_76_O
23	19.870	0.3	Digitoxin	C_41_H_64_O_13_
24	20.550	0.1	Oleic Acid	C_18_H_34_O_2_
25	21.021	0.2	1,25-Dihydroxyvitamin D3, TMS derivative	C_30_H_52_O_3_Si
26	21.386	0.3	Cucurbitacin b, 25-desacetoxy-	C_30_H_44_O_6_
27	21.681	1.0	2-Myristynoyl pantetheine	C_25_H_44_N_2_O_5_S

**Table 5:** Table showing the % of cells expressing Cyt C in Untreated, Std, and *S. lappa*-treated HepG2 cells.

Cells Group 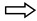 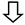	% of Cells Expressed Cytochrome C
Cell Control	0.64
Positive Control	82.89
*S. lappa*	67.78

## Data Availability

The raw data used and/or analyzed during the current study will be available from the corresponding author on reasonable request.

## Supplementary Materials

The following are available online at https://www.mdpi.com/article/10.3390/cimb43020079/s1. Figure S1: Agarose gel electrophoresis image of Isolated RNA, Figure S2: Validation of primers with each cDNA, Figure S3 Melting curves of genes. Melting temperatures were visualized by plotting the negative first derivative of fluorescence relative to the temperature in Celsius [-(d/dT)] and Figure S4: Amplification curve.
