# Supplementary material for: Saussurea lappa Exhibits Anti-Oncogenic Effect in Hepatocellular Carcinoma, HepG2 Cancer Cell Line by Bcl-2 Mediated Apoptotic Pathway and Mitochondrial Cytochrome C Release
Source: Curr Issues Mol Biol. 2021 Sep 8;43(2):1114–32. doi: 10.3390/cimb43020079 (PMC8929068; doi:10.3390/cimb43020079)
Supplement: Supplementary file 1 [file cimb-43-00079-s001.zip › cimb-1362012-supplementary.pdf]

UT *S.lappa E* Camptothecin 100bp ladder

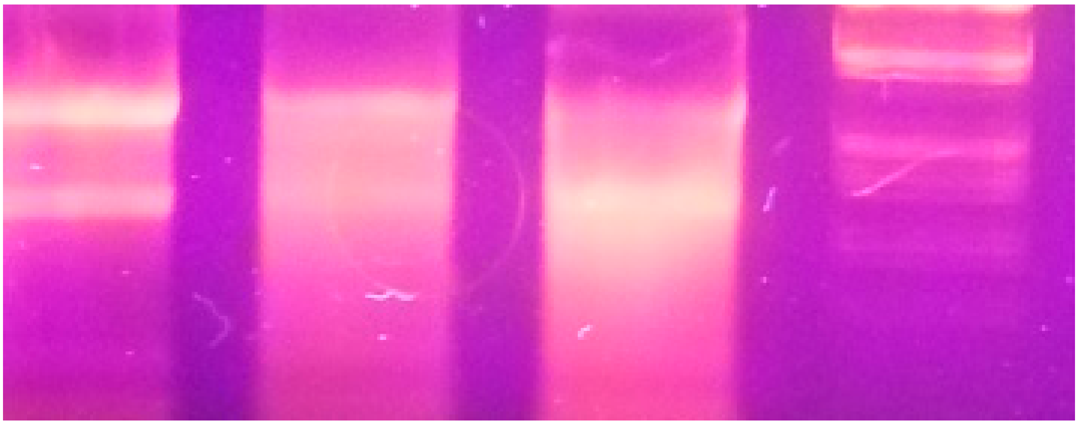

Fig S1: Agarose gel electrophoresis image of Isolated RNA

Primer Validation

All 3 gene specific primers and a house keeping gene primer were validated by PCR using mixed pool of cDNA from Normal and cancer tissues. The amplicons were analysed on 2% agarose gel along with 100bp ladder with bright band at 500bp. Primers were then validate with SYBR reactions for amplification and melt curves.

All the primers were amplified the expected size product with no self-annealing or self-dimerization property.

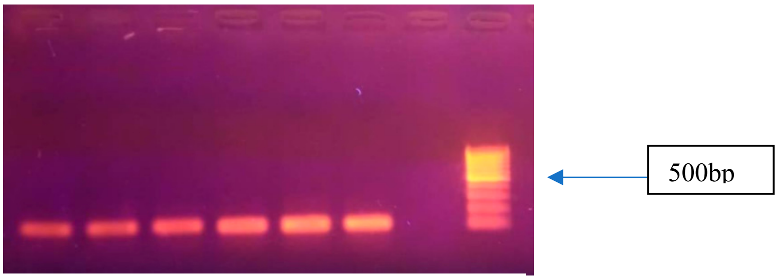

Caspase3 Bcl2 Bax Ladder

Fig S2: Validation of primers with each cDNA

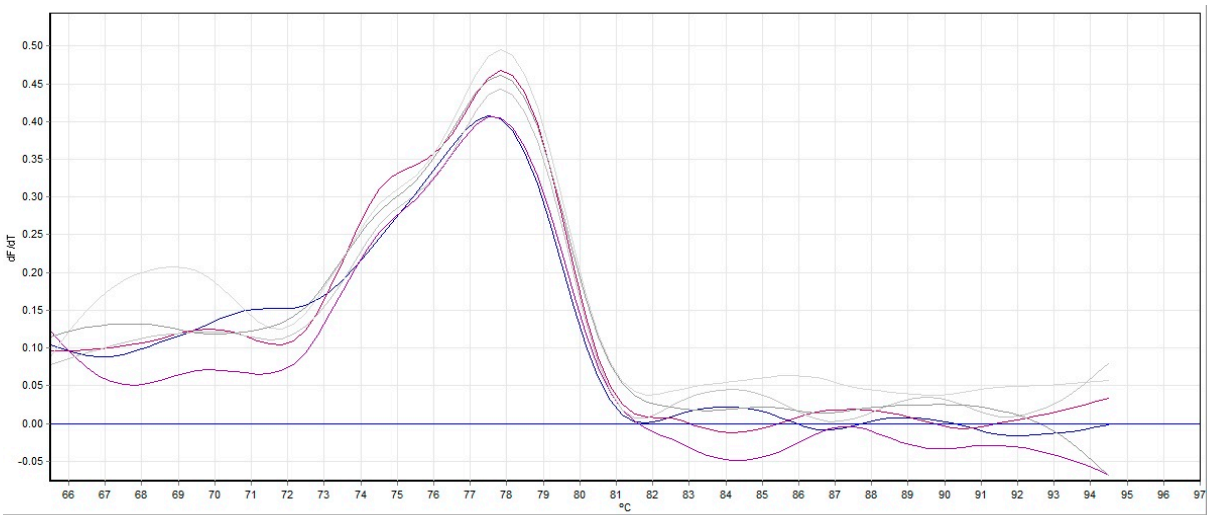

Caspase-3

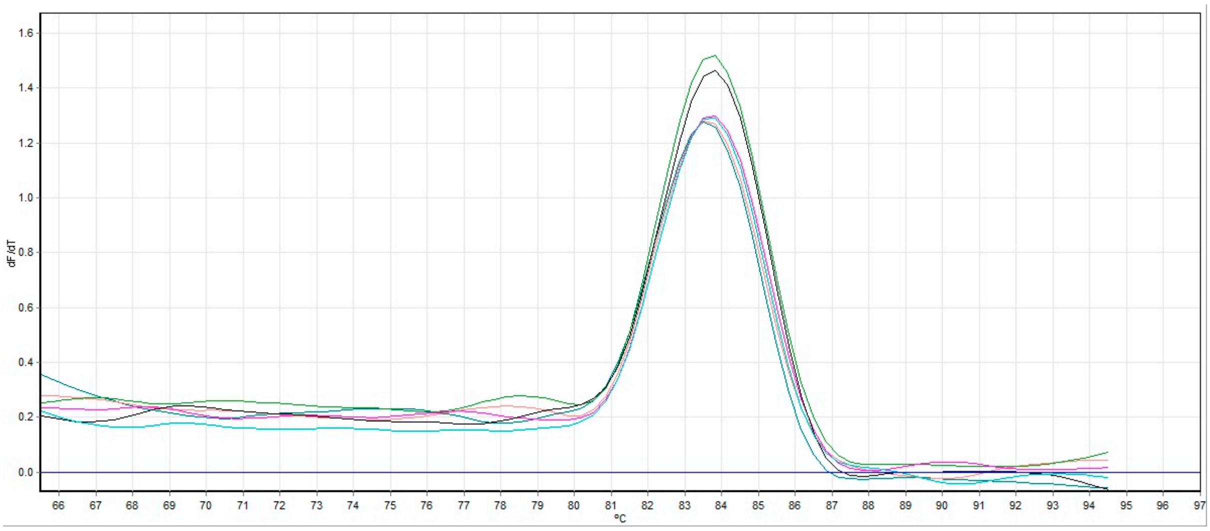

Bcl-2

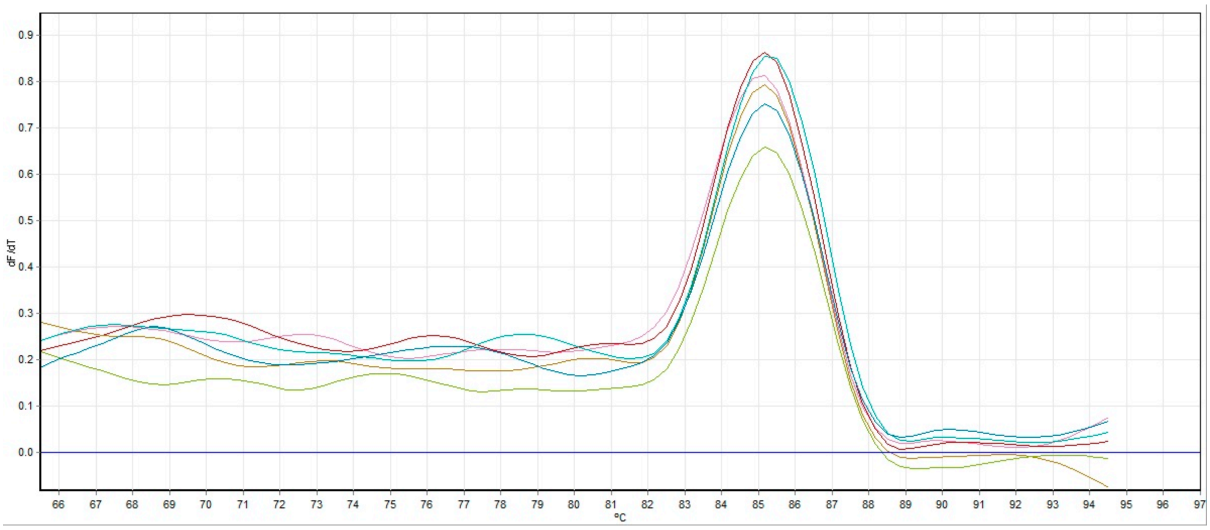

Bax

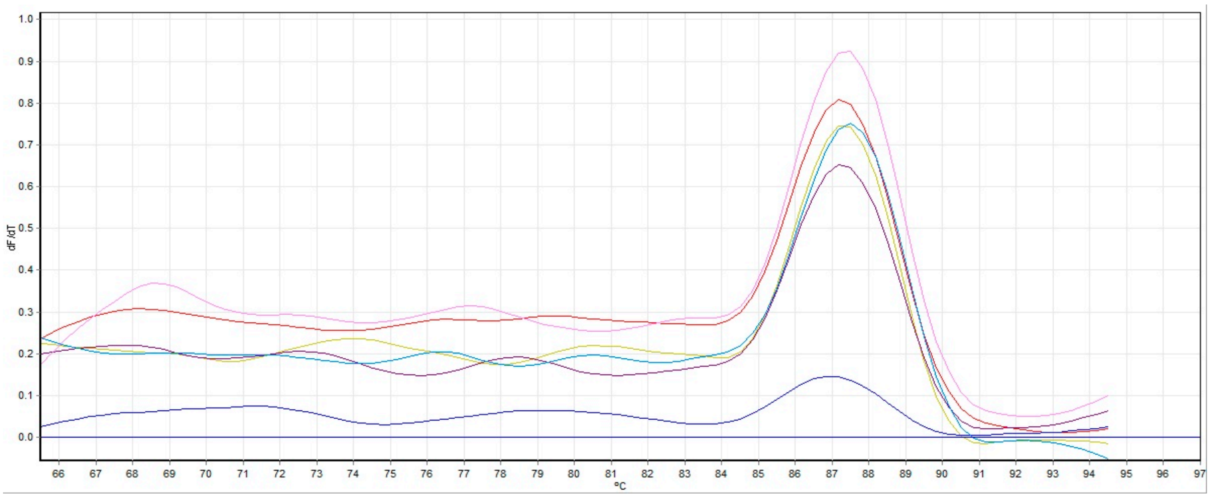

GAPDH

Fig. S3 Melting curves of genes. Melting temperatures were visualized by plotting the negative first derivative of fluorescence relative to the temperature in Celsius [-(d/dT)].

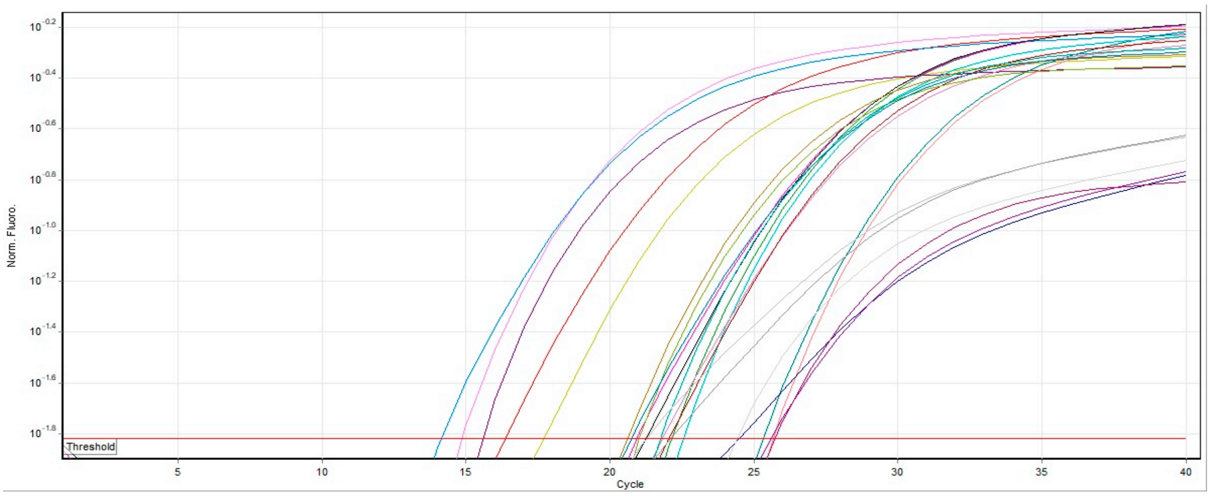

Fig. S4: Amplification curve
